# Repeated Thermomechanical Recycling of Polypropylene-Organosheets to Injection-Moulded Glass-Fibre-Reinforced Composites

**DOI:** 10.3390/polym17182528

**Published:** 2025-09-18

**Authors:** Barbara Liedl, Thomas Höftberger, Gernot Zitzenbacher, Christoph Burgstaller

**Affiliations:** 1 Transfercenter für Kunststofftechnik GmbH, Franz-Fritsch-Str. 11, 4600 Wels, Austria; barbara.liedl@tckt.at (B.L.); thomas.hoeftberger@tckt.at (T.H.); 2 School of Engineering, University of Applied Sciences Upper Austria, Stelzhamerstr. 23, 4600 Wels, Austria; gernot.zitzenbacher@fh-wels.at

**Keywords:** mechanical recycling, injection moulding, glass fibre

## Abstract

Continuous-fibre-reinforced thermoplastics are attractive materials for industries to cut down on weight in structural components. Recycling these parts or trims generated during production is difficult due to the reduced properties in materials intended for high-performance applications. Our study investigates the recyclability of short-fibre-reinforced compounds made from shredded organosheets. The fibre share was varied by the addition of virgin polypropylene, and three recycling rounds via a reduced injection-moulding process and a full thermomechanical recycling process including a compounding step were compared. Organosheet cuttings were found to be able to be applied as a short-glass-fibre source for the production of composites with varying fibre shares. Up to 14,000 MPa of elastic modulus and 80 MPa of tensile strength could be achieved at a fibre content of 45 vol%. Fibre length was reduced with progressive processing, less so for lower fibre shares, and in the reduced process without the shear and stress of the compounding step. Fibres from organosheets might be present in bundles and disperse in the matrix with progressive processing, which is particularly the case without compounding processes and can also influence the mechanical properties.

## 1. Introduction

Fibre-reinforced plastics have a wide range of applications due to their diversity in composition and their outstanding properties. Integrating fibres into a polymer matrix enhances mechanical properties and opens up opportunities for light-weight construction of structural components, e.g., in automotive, aerospace, or leisure industries. The market for these so-called composite materials is constantly growing. Various types of fibres are used in composites, but glass-fibre-reinforced composites make up the largest share of the market, due to their good price–performance ratio [1]. In 2019, this share was 88%, followed by natural fibres with 11% and carbon fibres with 1% of the market share [2]. In 2015, the global production of glass-fibre-reinforced composites was indicated to be more than 10 million tons per year [3].

The term fibre-reinforced composites (FRCs) includes a variety of materials, which not only differ in the type and length of fibre, but probably most decisively, in the type of matrix material used: thermosets or thermoplastics [4]. Although thermoset composites are more common with a market share of 61% compared to thermoplastic composites with 39% in 2019, there are several advantages to using the latter: shorter mould times, better impact absorption, the possibility to store semi-finished products at room temperature, unlimited shelf life, and short cycle times, to name a few. Whereas thermosets are fully crosslinked resins, thermoplastics can be remelted and hence also offer other recycling possibilities [2,5,6]. Thermoplastic-matrix composites are further divided into continuous- and discontinuous-fibre-reinforced composites, according to the length and nature of their reinforcement. Continuous-fibre-reinforced plastics are either reinforced with unidirectional tapes, wovens, or others, like braided or knitted mats. Discontinuous-fibre-reinforced composites are classified according to the aspect ratio of the fibres (length/diameter) into long- or short-fibre composites [6]. The effort to produce continuous-fibre-reinforced composites is incomparably higher than the production of short-fibre-reinforced composites. The first needs high-level automation processes compared to the latter, and is mixed in melt processing with both heat and shear [7]. Even if the use of fibre-reinforced composites is basically intended for long and durable applications, the end-of-life issue is gaining increasing attention. With increasing composite use and thus increasing proportions of composite waste, the importance of finding valorisation options for these waste fractions rises. There is only an estimate of the amount of fibre-reinforced composite waste. About 0.5–1 million tons of waste is generated during the production of parts, wherefrom 70% is thermoset. The waste generated during production is currently estimated to be even higher than the waste of products reaching the end of their life, although the latter is more likely to increase in the future [2,3].

Recycling strategies for fibre-reinforced composites in general involve mechanical, thermal, and chemical approaches [8]. To only recover the fibres, whole composites can be grinded, and the ground particles can be separated to fibre-rich and matrix-rich fractions. Another approach is to thermally degrade the matrix into gases, oil, or energy, and in this way extract the fibres. Chemical recycling of fibre-reinforced materials is not very widespread, but it includes the depolymerisation or dissolution of the matrix phase to regain the fibres and potentially monomers and oligomers from the polymer. The recovery of glass fibres is less interesting than those of carbon fibres due to their large prize difference and loss in properties [9]. Glass fibres usually are mechanically recycled, whereas thermal recovery of the fibres is preferred for carbon fibres [1,2]. The higher commercial value of carbon fibres also explains the difference in research focus between these fibre types. Although glass fibres account for 88% of all composites, this distribution in quantity is not found in published research, but rather there is a strong focus on carbon-fibre-reinforced composites [2]. The problem in recycling glass fibres thermally is that they are not competitive (price–performance ratio) with new glass fibres due to their mechanical properties, some of which are greatly reduced. The high temperatures in thermal recycling lead to a loss of strength of the glass fibres [3]. While recycling of thermoset composites is limited to reuse of grinded material as filler in various applications, and possibly the elaborate removal of the fibres from the matrix, due to the cross-linked nature of thermosets, there is another option for recycling fibre-reinforced thermoplastics due to the re-meltability of the matrix. In general, shredding and reprocessing of the fragments is a common method in recycling thermoplastics [10]. However, with fibre-reinforced thermoplastics, shredding and reprocessing affects not only the polymer [11,12], but also the filler [13].

Recycling of thermoplastic composites, especially those reinforced with fibres, poses a challenge due to several issues. High costs, difficult market entry for recycled materials, and degrading properties are identified as the largest obstacles [2]. The latter is an especially inherent problem in recycling FRCs, as these types of material are intended for high-performance applications [13]. Long- and continuous fibre-reinforced plastics with a directional fibre structure and high fibre content, to which the organosheet used in this study belong, are high-performance materials. They are readily used in the automotive and aerospace industries, standing out with their crash absorption behaviour [1,14]. Even if the production wastes generated during the manufacturing of these parts can no longer be used for the same purpose, they still can potentially be recycled into discontinuous-fibre-reinforced thermoplastics. Short-fibre polypropylene (PP) composites are especially attractive materials, not only due to their mechanical properties, but also their beneficial price–performance balance [15,16]. Processing FRCs as whole in a thermomechanical recycling process, including shredding, extrusion to granules, and finally injection moulding to a new part, exposes the material to heavy stresses. Each step leads to renewable stress on the matrix and especially on the fibres. The most prominent factor contributing to performance decreases in processing fibre-reinforced thermoplastics with polymer processing machinery is the degradation of fibre length. Flow speed, shear forces, and equipment and mould geometry make fibres buckle up and break [7,17,18,19,20,21]. As the performance of a fibre composite is related to the fibre length, fibre length degradation inevitably leads to lower mechanical properties [2,15,22]. Also, the matrix polymers are degraded during repeated processing and chain scission can occur [23]. In multiple process cycles, the effect is multiplied by each round the material goes through. Several studies have dealt with recycling glass-fibre-reinforced thermoplastics, either using crushed parts of continuous-fibre-reinforced thermoplastics, or short-fibre composites. Glass-fibre-reinforced PP composite waste was successfully used to produce short-fibre filaments for additive manufacturing, blended with PP to varying fibre shares. Repeated recycling reduced the fibre aspect ratio, which in this case was beneficial for filament quality [24]. Glass-fibre-reinforced polyamide-6 organosheets were chopped and the choppings were used to produce compression-moulded sheets again [25].

**Figure 1:**
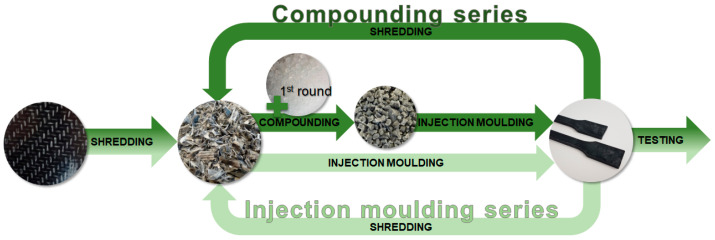
Process scheme for our recycling study: shredded organosheets (glass fibres in black coloured polypropylene matrix) were either compounded with or without additional PP in the first round to granules, and further injection moulded to test specimens. In the second and third process cycle, the shredded test specimens were compounded as they were and injection moulded (dark green). This process was compared to a process cycle without a compounding step and without additional PP added (light green).

Recycling of shredded PP glass-fibre and polyamide-6 carbon-fibre organosheets via compression moulding was demonstrated by Kiss et al. [26]. Where simply shredded and compressed laminates only achieved 10–30% of the strength of the initial material, this could be improved to 50% by using the chopped materials as a sandwich-like layer between intact fibre laminates. In addition to the other processing methods stated in the last preceding paragraph, glass-fibre-reinforced thermoplastics are recycled by injection moulding, as we did in our study: PP reinforced with 40 wt.% glass fibre (GF) of >1 mm in length was injection moulded, grinded, and mechanically recycled by Colucci et al. The average fibre length decreased after recycling the composites, as well as the E-modulus and tensile strength. Nevertheless, once-recycled 40 wt.% GF-PP still gave high-performance properties [27]. Fibre length and hence performance degradation was also shown for recycling polyamide 6,6 glass-fibre composites via injection moulding and granulation [28]. Multiple recycling sequences for fibre-reinforced PP was rather published for natural-fibre-reinforced PP [29,30]. Evens et al. investigated the effect of ten recycling sequences in a comprehensive study with unfilled polypropylene, and PP filled with glass fibres, carbon fibres, and flax fibres. The fibre content was 20 wt.% each. It was shown that multiple recycling sequences has a fundamental detrimental effect on the mechanical properties of fibre-reinforced PP, though there was an increase in impact strength for flax- and carbon-fibre composites. The materials were injection moulded and shredded tenfold, but no extrusion step was performed before the injection moulding as in our study. Also, the investigated materials were not organosheets, but short-fibre-reinforced composites [13]. We believe that extrusion is often a necessary step, as the shredded material (as depicted in Figure 1) is not free flowing, and is complicated to handle in processing.

The aim of our work was to examine repeated processing of polypropylene–glass-fibre composites, starting from a roving glass composite laminate (organosheet). Three consecutive processing cycles were performed to imitate a multiple mechanical recycling process. Our approach was to shred the organosheet and implement the shredded particles—containing chopped glass fibres—as reinforcement for newly produced glass-fibre-reinforced composites. We varied the glass-fibre content by the addition of polypropylene in the compounding step. Although composites with a high performance can only be obtained with a high fibre concentration [31], blending with matrix polymer is an option to produce materials that are still interesting. Moreover, we compared two processes—a complete thermoplastic processing cycle including extrusion to granules and injection moulding and a reduced processing with only the glass-fibre composite being injection moulded, which was not reported on previously for organosheets. We evaluated if the shredded organosheet is suitable for use as a reinforcing material and glass-fibre source for PP composites, and how the materials produced from it and their fibre lengths and mechanical properties are affected by multiple thermomechanical recycling processes.

## 2. Materials and Methods

### 2.1. Materials and Preprocessing

The organosheet we used in our study was a roving glass–PP consolidated composite laminate from Lanxess (Cologne, Germany) named Tepex^®^ dynalite 104-RG600(x)/47%. The datasheet indicates a nominal fibre content of 47 vol.%. The actual fibre content of the organosheet was determined by thermogravimetric analysis in a macro TGA 701 from LECO (St. Joseph, MI, USA) according to ISO-3451 [32] with a calcination temperature of 625 °C. Two test samples of about 1 g each were measured, and the fibre weight fraction was determined to be 69.60 ± 0.15 wt.%. The density of the organosheet was measured according to ISO-1183 [33], using three replicates of pieces cut out and weighing them in air and immersed in ethanol on an AX224 balance from Sartorius, Germany equipped with a density kit YDK01. The fibre volume content V_f_ was calculated using Equation (1), with the density of the organosheet (ρ_c_), the density of the matrix (ρ_m_, 0.908 g/cm^3^), and the density of the fibre (ρ_f_, 2.54 g/cm^3^). This formula is simply deducted from the fibre volume content definition, with the definition of the density being equal to mass divided by volume. The fibre volume share of the organosheet was calculated to be 45.01 ± 0.10 vol.%. For the sake of simplicity, we continue to use the value rounded to 45 vol.% in the following sections.

(1) $$V_{f}=\frac{\rho_{c}-\rho_{m}}{\rho_{f}-\rho_{m}}$$

The organosheet was cut to pieces of about 10 × 10 cm with a conventional band saw and shredded to pieces on a cutting mill (MAS1, Wittmann, Vienna, Austria) equipped with a 4 mm screen to exclude particles above this size. The bulk density of the shredded material was determined in triple determination by weighing a 100 mL vessel completely filled with the shredded pieces. The bulk density of the shredded organosheet was 0.44 ± 0.00 g/cm^3^. In comparison to that, the bulk density of the PP granules was 0.70 ± 0.00 g/cm^3^.

### 2.2. Recycling Processing Methods

The shredded organosheet was processed in several ways: on one hand, the shredded pieces were directly processed into universal test specimens on an injection moulding machine, and on the other hand, they were extruded to granules in a twin-screw extruder. Two mixtures were also made in the extrusion process: compounds with an intended fibre share of 30 and 15 vol.% were produced by blending the shredded organosheet with polypropylene (HE 125MO from Borealis, Linz, Austria) in the extruder. The so-produced granules were consequently also injection moulded to universal test specimens. A multiple recycling process was simulated by reprocessing the materials after injection moulding: the sprues, runners, and surplus test specimens not required for characterisation were again shredded by the cutting mill to pieces of less than 4 mm in size and subsequently processed like they were already treated in the first round. This was repeated two times, to ultimately have three materials of each composition: once, twice, and thrice processed. An overview of the materials processed in this study is given in Table 1, named after the intended glass-fibre content, the processing round, and the methods used. A scheme of the process is presented in Figure 1.

Extrusion was performed in a co-rotating twin-screw extruder (TSE 24 MC, ThermoFisher Scientific, Karlsruhe, Germany) having a screw diameter of 24 mm and a screw length of 40 L/D. The temperature profile and screw configuration are shown in Table 2. Strands were produced with a three-hole die and cooled down in a water bath, and granules of about 3 mm in length were chopped with a strand granulator. In the first round, the granules and the ground organosheet for the injection moulding series were dried in a dry-air dryer at 90 °C for 1 h before all the material was injection moulded to universal test specimens on an injection moulding machine (Victory 80, Engel, Schwertberg, Austria). The mass temperature for all compounds was 230 °C, the tool temperature 40 °C, the dosing volume 45 cm^3^, and the dynamic pressure 150 bar.

### 2.3. Characterisation Methods

The test specimens for mechanical testing were put aside and stored in a climate chamber (KBF 720, Binder, Tuttlingen, Germany) at standard climate (230 °C, 50% r.h.) for at least 96 h until testing according to ISO-291 [34]. The rest of the test specimens together with the sprues and the runners were shredded using a 4 mm screen on a cutting mill (MAS 1, Wittman, Vienna, Austria) and used as input material for the repetition of the respective recycling process.

The fibre length of the compounds after each completed recycling cycle was measured using the residue of universal test specimen after thermogravimetric analyses (TGA 701 from LECO, St. Joseph, MI, USA) at a calcination temperature of 625 °C. Microscope images were taken of the recovered glass fibres put on a microscope slide with an Olympus BX 61 with a DP27 microscope camera (Olympus Europa SE & Co. KG, Hamburg, Germany). The lengths of 600–1500 individual fibres for each sample were measured. The fibres were clustered into classes according to their length and prevalence. The length-weighted fibre length was calculated (by summing up the fractions of each class’s sum of the average fibre length times the number of fibres in the class, divided by the sum of the average fibre length squared times the number of fibres in the same class), to give less weight to fragments split into many small pieces. Through this length-weighted calculation and the large number of fibres in the calculation, giving a standard deviation of the fibre length is superfluous.

Density of the compounds was measured according to ISO-1183 [33] using parts of universal test specimens. Five replicates each were measured on an AX224 balance from Sartorius, Germany, equipped with a density kit YDK01.

Porosity of the test specimens was determined with the actual measured density and the theoretical density using Equation (2). The actual density was measured as described above. Two replicates each were measured on an AX224 balance from Sartorius, Germany, equipped with a density kit YDK01. These two samples were then calcinated on a macro TGA 701 from LECO (St. Joseph, MI, USA) according to ISO-3451 [32] to determine the actual fibre content and hence calculate the theoretical bulk density of the specimen with absence of any pores, using the densities of the individual components, e-glass fibre (2.54 g/cm^3^ according to literature [35,36]), and PP (0.908 g/cm^3^ according to the datasheet of HE 125MO).

(2) $$Porosity\;\left[\%\right]=\left(1-\frac{\rho_{measured}}{\rho_{calculated}}\right)\cdot 100$$

Melt flow rate (MFR) was measured according to ISO-1133 [37] on a flow test device (4106, Zwick-Roell, Ulm, Germany) with three replicates, using test specimens shredded to less than 4 mm in size on the cutting mill. The materials were dried before the measurement in a drying cabinet UF55 from Memmert GmbH + Co. KG (Schwabach, Germany) for 1 h at 90 °C.

Tensile tests were carried out on a 20 kN universal test machine Z020 (Zwick-Roell, Ulm, Germany) with five replicates. For the determination of the elastic modulus, the testing speed was set to 1 mm/min in the first 0.25% of elongation followed by a speed of 5 mm/min until test specimen failure in accordance with ISO-527 [38].

Impact strength and notched impact strength was measured according to ISO-179 [39] on a pendulum impact test device (5113.300, Zwick-Roell, Ulm, Germany) with a set of ten replicates each. The test specimens were cut out from universal test specimens to a size of 80 × 10 × 4 mm; for the notched samples, the V-notch Type A (0.25 mm radius, 45 ± 1° angle, 2 mm depth) was cut on a precision notch saw (Mutronic Diadisc 4200, Rieden am Forggensee, Germany).

## 3. Results and Discussion

PP–glass-fibre organosheets were shredded and processed in two ways to represent multiple recycling sequences applied to short-fibre-reinforced composites. One series was done, representing a whole processing chain, by extruding the organosheet pieces in a twin-screw compounder, both as pure pieces and with additional PP, to obtain different fibre contents. The granules produced in this way were injection moulded into test specimens, shredded, and reprocessed twice. The second series was done to compare the process to a reduced processing chain, without the compounding step. The chopped organosheet was injection moulded into test specimens, shredded, and reprocessed two times.

### 3.1. Comparing Fibre Content

**Figure 2:**
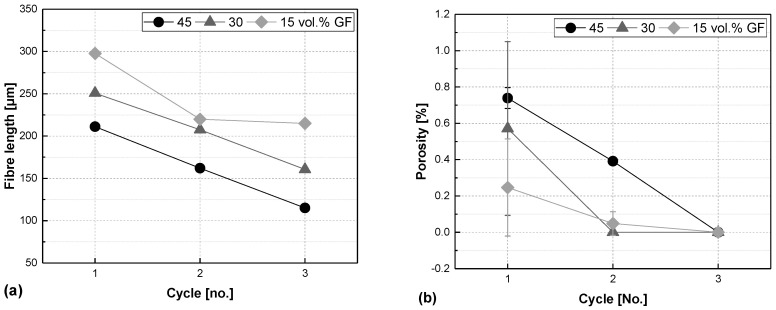
Fibre length (**a**) and porosity (**b**) vs. processing cycle number of glass-fibre-reinforced composites made from organosheets with a fibre share of 45, 30, and 15 vol.% (Remark: Some error bars given in the figure are smaller than the symbol size).

The average fibre length determined for the shredded organosheet of less than 4 mm in size was 660 µm. After compounding and injection moulding, the fibre length decreased for all samples almost linearly with progressive processing, as shown in Figure 2 (left). The lower the share of shredded organosheet and hence glass-fibre content in the final compound, the longer the fibres. Glass fibres in GF30 preserve almost 50 µm more of their fibre length compared to GF45 with no additional PP added. Another 50 µm of fibre length is preserved in GF15 samples compared to GF30, though there is a little drop in round two. Nonetheless, glass fibre length is degraded strongly over the entire process and for all of the compounds, ending up with fibres of 115 µm (GF45-R3), 160 µm (GF30-R3), and 215 µm (GF15-R3) after a total of three processing cycles.

The porosity measured for the plain organosheet is 0.85 ± 0.05%. Figure 2 clearly shows the trend for the compounds produced: the higher the fibre volume content, and the less advanced the processing is, the higher the is porosity. The standard deviation of some samples might be high due to inhomogeneous spreading of the fibres.

**Figure 3:**
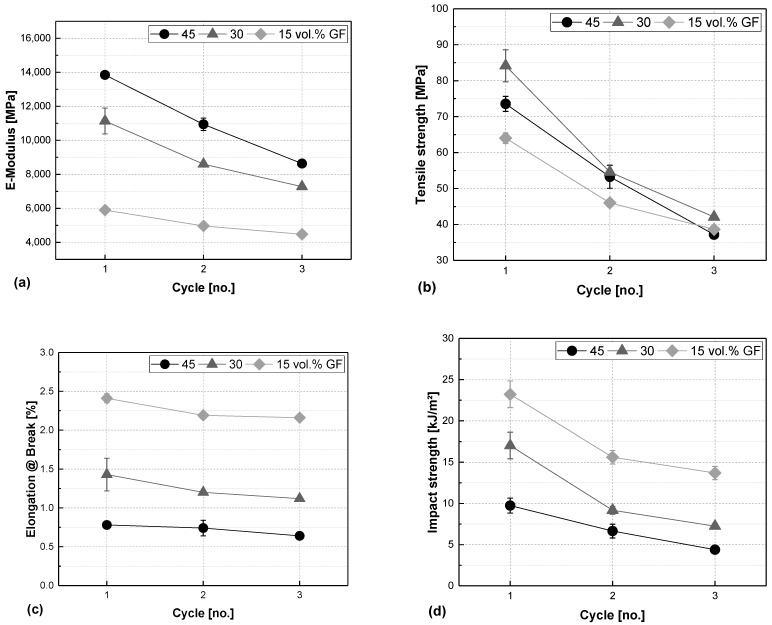
Elastic modulus (**a**), tensile strength (**b**), elongation at break (**c**), and unnotched impact strength (**d**) vs. processing cycle number of glass-fibre-reinforced composites made from organosheets with a fibre share of 45, 30, and 15 vol.%. (Remark: Some error bars given in the figure are smaller than the symbol size).

Figure 3 shows the results of the tensile tests and the impact strength. The E-Modulus is generally lower for lower fibre shares and decreases over the processing cycles for all three fibre shares. The decrease with fibre length and fibre content is in good accordance with the literature [22]. Also, tensile strength is less dependent on the fibre volume content, even reaching higher values for the material containing 30 vol.% glass fibres in the first processing round, but is rather influenced by fibre length [40]. Strength decreases drastically for all materials, finally reaching a value of around 40 MPa after processing round 3, which accounts for a reduction of almost half of the initial strength. Elongation at break is influenced by fibre content rather than fibre length, although there is a slight decrease in the values from processing round 1 to round 3. The compounds with higher fibre shares exhibit lower elongations at their breaking point compared to the materials with a lower fibre content. As in elongation at break, impact strength is also determined by the fibre content. For the plain organosheet GF45, this decrease is linear over the reprocessing, and the materials with lower fibre shares, such as GF30 and GF15, exhibit a drop from cycle 1 to cycle 2. Fibres act as fault locations leading to easier breakage of the material at abrupt impact loads, especially when they are not perfectly connected to the matrix.

We used the modified Kelly–Tyson equation (Equation (3)) for composites with subcritical fibres [29,41,42] to assess the bonding of the fibres to the matrix. Interfacial shear strength (IFSS, τ_c_) was calculated for all compounds produced. The strength of the composite (σ_c_) was determined by tensile tests as well as the reduced tensile strength of the matrix at tensile strain of the composites (σ′_m_). The fibre orientation factor (η_O_) was assumed to be 0.75 [42,43]. The fibre diameter (d, 19 µm) as well as the length of the fibres (l) were determined by microscopic measurements as described above. The fibre volume fraction (V_f_) is known from thermogravimetric analyses.

(3) $$\sigma_{c}=\eta_{O}\tau_{c}\frac{l}{d}V_{f}+\sigma_{\;m}^{\prime }(1-V_{f})$$

**Figure 4:**
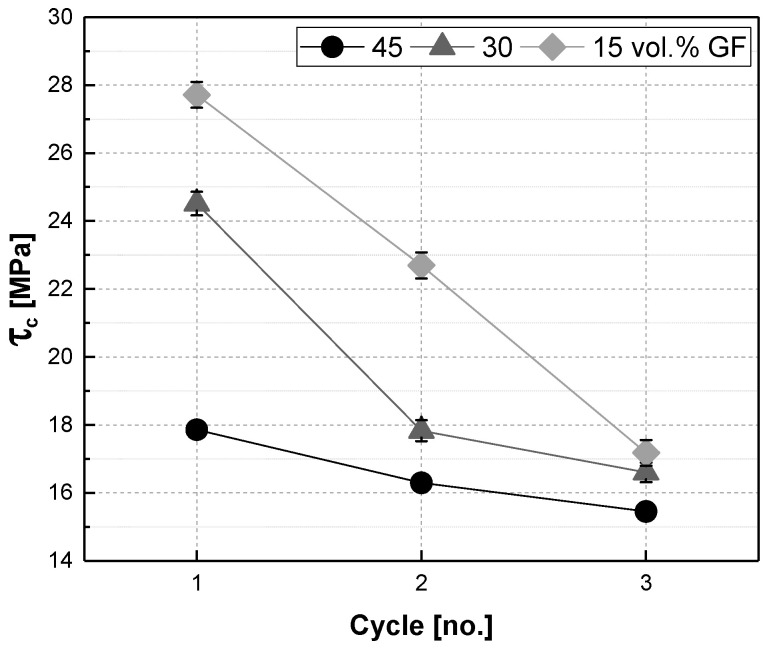
Interfacial shear strength (τ_c_) values calculated with a modified Kelly–Tyson equation vs. processing cycle number for glass-fibre-reinforced composites made from organosheets with a fibre share of 45, 30, and 15 vol.% (Remark: Some error bars given in the figure are smaller than the symbol size.).

The fibre content is constant over the processing cycles, and interfacial shear strength is expected to remain constant too. Despite this, Figure 4 reveals the opposite: the calculated values for IFSS drastically decrease with progressive processing. This effect is most evident for GF15, which generally has the highest IFSS in round 1, followed by GF30 and GF45 with the lowest IFSS. For all compounds, the IFSS is decreasing almost linearly and the values for all three compounds converge in round 3 around 15.5–17.5 MPa. The IFSS for GF15 is nearly halved from cycle 1 to cycle 3, whereas the material with the highest fibre content, GF45, is only exposed to a smaller decrease in IFSS.

**Figure 5:**
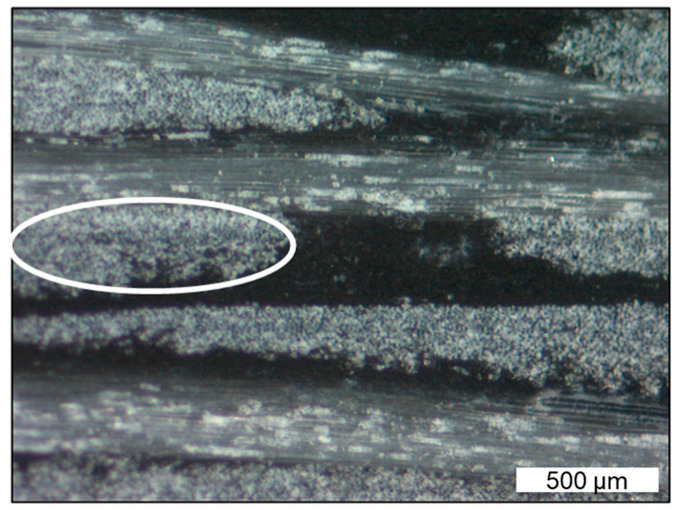
Micrograph of an organosheet cross-section, 40 times magnified, with the layers of the glass-fibre rovings going in both directions (parallel to the picture and coming out of it). One fibre bundle, consisting of several hundred single glass-fibre strands, coming out of the plane of the picture is marked with a white ellipse.

IFSS is influenced by several factors, such as the crystallinity of the matrix [44]. It could significantly be improved by the addition of coupling agents, such as silane or maleic acid anhydride functions [45,46,47]. Although not stated in the data sheet, it can be assumed that there are some adherence modifiers applied in the organosheet. Nonetheless, we did not add any additional coupling agents. Our assumption for the decrease in IFSS over the processing cycles, which is much more severe for lower fibre contents, lies in the nature of the fibres in the fractions of organosheet we used. Figure 5 shows the cross-section of the organosheet, where the fibres are arranged in tight bundles in the fibre mat. These fibre bundles are assumed to break up due to shear during processing, breaking up a little more in each round, and faster at lower fibre contents. Due to the resulting increase in surface area between the fibres and matrix, and the lack of a coupling agent, the attachment between the fibres and matrix is decreasing, resulting in a lower IFSS.

### 3.2. Comparing Processing Routes

A series with an exclusive injection moulding process (IM) was carried out to be compared to the series including compounding (C+IM) described above. It is assumed that fibre length decreases less when only injection moulding occurs, as the material is much less exposed to shear due to the lack of a processing step.

**Figure 6:**
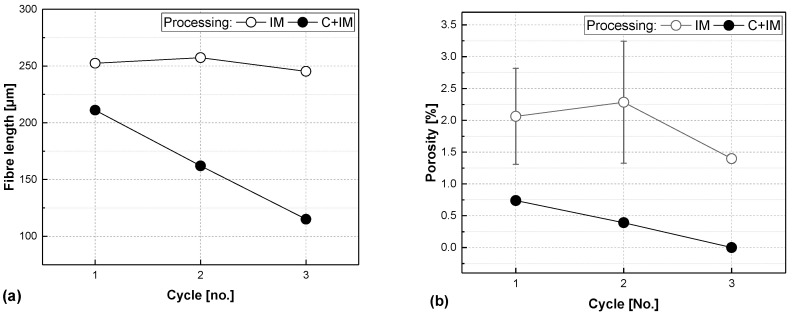
Fibre length (**a**) and porosity (**b**) vs. processing cycle number of glass-fibre-reinforced composites made from organosheets by injection moulding (IM) or additional compounding before injection moulding (C+IM) with a fibre share of 45 vol.%. (Remark: Some error bars given in the figure are smaller than the symbol size).

Figure 6 proves this assumption correct: the fibre length in the IM series is largely preserved over all three processing cycles, although there is a large difference to the initial fibre length of the shredded organosheet (660 µm). The C+IM series stands in contrast to this, where the fibres are already shorter than in the IM series after the first round of processing, and moreover linearly decrease to almost half of their initial length after cycle 3.

Porosity is high for the injection-moulded samples, and with a value of 2.06 ± 0.76%, it is a lot higher than for the plain organosheet (0.85 ± 0.05%). For the IM samples, there is no clear trend in decreasing porosity, as it is for the C+IM samples. The standard deviation for the IM samples is also high, which could be due to inhomogeneous spreading of the fibres throughout the specimen.

**Figure 7:**
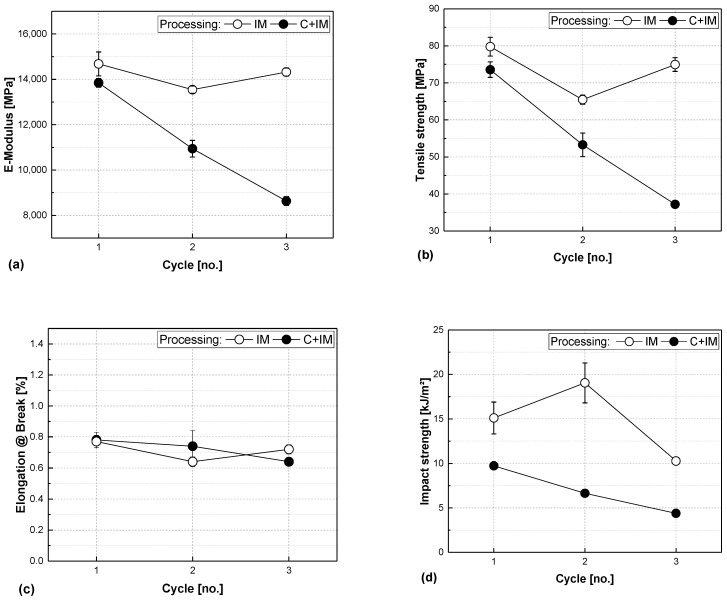
Elastic modulus (**a**), tensile strength (**b**), elongation at break (**c**), and unnotched impact strength (**d**) vs. processing cycle number of glass-fibre-reinforced composites made from organosheets either by injection moulding (IM) or additional compounding before injection moulding (C+IM) with a fibre share of 45 vol.%. (Remark: Some error bars given in the figure are smaller than the symbol size).

The results of the tensile test and impact strength are compared in Figure 7. For all measured parameters, C+IM samples exhibit a more or less linear decrease. The values for E-modulus, tensile strength, and impact strength are almost halved from processing round 1 to processing round 3. The IM series behaves quite differently: for the E-modulus, tensile strength, and elongation at break, there is also a drop from round 1 to round 2. However, after that, the values rise again in round 3 instead of continuing to fall as in the C+IM series. In all cases, the values of round 3 remain slightly below the initial round 1 values. Impact strength decreases more sharply from round 1 to processing round 3 by about a third of the values. However, the samples of cycle 2 show a peak in impact strength that is even higher than the initial value in cycle 1.

**Figure 8:**
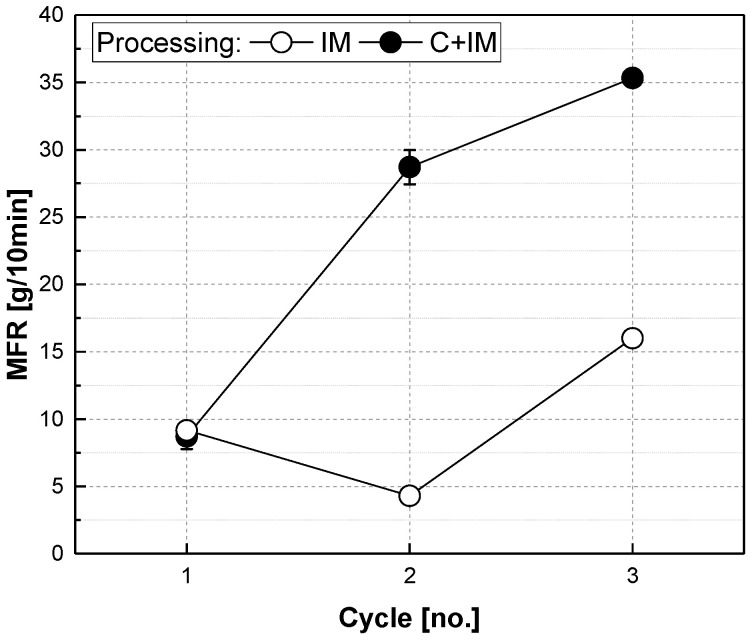
Melt flow rate (MFR) of glass-fibre-reinforced composites made from organosheets either by injection moulding (IM) or additional compounding before injection moulding (C+IM) with 45 vol.% fibre share. (Remark: Some error bars given in the figure are smaller than the symbol size).

Measurements of the melt flow rate in Figure 8 show a similar trend to the mechanical tests. Whereas the MFR of the C+IM series increases almost linearly with progressing cycles, most likely due to the PP matrix degrading in the process and the fibre length reductions, the values for the IM series rise, but also have a familiar deviation in round 2, which can most likely be attributed to the improved dispersion of the fibre bundles from the original organosheet.

### 3.3. Discussion

Repeated thermomechanical recycling of shredded PP–glass-fibre organosheets was shown. The results of our study can be divided into two main sections: the influence of the fibre content on the properties of the recycled materials produced, and the way or processing route of recycling (a reduced series with injection moulding only, and a full recycling cycle with compounding the materials before injection moulding).

The shredding and recycling of organosheets and the production of glass-fibre-reinforced PP composites with varying fibre shares led to a linear reduction in fibre lengths. The decrease in fibre length that occurs during the processing of rigid fibres is in accordance with the previous literature [7,17,18,19,20,21]. The porosity of the injection-moulded specimens was found to decrease with both fibre share and progressive processing steps. This trend makes sense for the fibre content, as less fibres in more matrix can cause less gaps. With shorter fibres, there might also be less overlap of fibres. There is something else to consider, as we did not use chopped short fibres for the production of our compounds, but shredded organosheets. In organosheets, the fibres are compressed together and impregnated with the matrix PP. This results in fibre bundles, which are not dispersed in the initial recycling step, as could be seen from our results. With progressing shear and an increased number of processing steps, these bundles break up and spread throughout the matrix. This could happen more quickly with lower fibre contents, as there is less fibre–fibre interaction to be expected. The calculation of the IFSS for the various fibre contents and the decrease in the IFSS over the recycling steps supports our thesis. As the fibre content, as well as the composite composition, does not change over the processing cycles and the processing temperatures are way below the degradation temperatures of the matrix and the sizing at the fibre–matrix interface, the IFSS is expected to stay constant. As this is not the case, we can only conclude that there is an increase in fibre–matrix interface, and hence a better dispersion of the fibre bundles, that is responsible for a decrease in the IFSS.

This becomes clearer when comparing the two processing methods: injection moulding (IM) applies less shear and the compounding + injection moulding (C+IM) method adds more shear stress. The lower shear stress is immediately apparent in the fibre length, which is largely preserved for the IM series. The porosity however, as indicator for bundles present in injection-moulded samples, is a lot higher for the IM series than for the compounded ones, also supporting our findings.

The general trends we found in our study are in good accordance with results found from other researchers working on glass-fibre-reinforced materials. With a decrease in fibre length, the mechanical properties also decrease. In our case, after one recycling step including only injection moulding, we found a tensile strength of approx. 80 MPa, which is in good accordance with results from another work on a single injection moulding reprocessing step, where the authors found a value of about 90 MPa [48]. Comparing our results with a lower fibre content (i.e., 15 vol%) with the literature, we found a reduction in the elastic modulus from approx. 6000 to 4500 MPa and a reduction in the tensile strength from approx. 65 MPa to 40 MPa, which is in good accordance with the work from Evens et al. [13], who found a reduction in the flexural modulus from approx. 4000 to 3000 MPa, but a higher tensile strength (70 to 58 MPa), which can be attributed to processing by injection moulding only and the slight difference in fibre content. Another work from Achukuwu et al. shows much less degradation in multiple reprocessings of 20% of glass-fibre-reinforced polypropylene, i.e., from approx. 48 to 45 MPa, but the starting values were nonetheless much less due to the differences in composition [49].

This shows that the general trends are comparable, but the specific changes depend a lot on the composition of the glass-fibre-reinforced composites and the processing methods and parameters applied. Utilizing organosheet shreds as reinforcements is more comparable to long-glass-fibre reinforcements than regular glass fibres, which is also shown in the literature [48].

## 4. Conclusions

Shredded PP organosheets can be reprocessed to short-fibre-reinforced PP composites with a high fibre share of up to 45 vol.%. For lower fibre content composites, they can act as a source for short glass fibres. The nature of the fibres previously processed to organosheets has to be considered, as they are not loose or lightly bound together with the fibre sizing, but compressed to fibre bundles and coated with some matrix already, which may disperse only in the later stages of processing. Multiple processing of the compounds is possible for all investigated fibre shares from 15 to 45 vol%. Also, processing using either injection moulding only, or a combined compounding and injection moulding process, is possible, although fibre length suffers more in the latter. Despite decreasing fibre length, the mechanical properties still remain acceptable after recycling, especially after the first round of recycling, yielding nearly 15,000 MPa in elastic modulus and about 80 MPa of tensile strength for the composite with 45 vol.% of fibres. This makes not only organosheet trims attractive as a primary source for short-fibre-reinforced composites, but also shows that thermomechanical recycling is an option even for fibre-reinforced materials that are considered as non-recyclable.

Future investigations should focus on the fibre–matrix interface and the improvement of the binding to obtain even better characteristics. Furthermore, it would be interesting to examine other fibre–matrix systems.

## Figures and Tables

**Table 1:** Overview of compounds produced in our recycling study. (GFs—glass fibres, OS—organosheet, PP—polypropylene, GF—glass-fibre content, IM—injection moulding, and R denotes the recycling round).

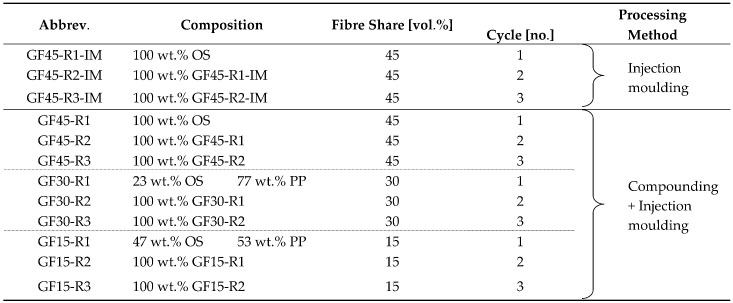

**Table 2:** Compounding temperatures (top line) and screw geometry (below). Each zone is about 4 L/D, and each kneading block element represents 0.25 L/D. At the intake and feed zone, 9 L/D conveying elements (c) ensure that the material is fed in. A kneading block in Zone 3 with 4 L/D melts and homogenizes the material. The material is transported over 11 L/D conveying elements to the next kneading block with a length of 3.25 L/D. This kneading block has the purpose to homogenize the material and mix the fibres with the polymer melt. Another zone of 4 L/D conveying elements follows before the last kneading block with 2 L/D and the same purpose of mixing the material. A total of 5 L/D conveying elements and 1.75 extrusion discharge elements to build up pressure and achieve a constant output finish the extruder configuration were used.

**Extruder Zones**	**Intake**	**Zone 2**	**Zone 3**	**Zone 4**	**Zone 5**	**Zone 6**
Temperature [°C]	40	190	200	200	200	200
Screw elements	c	c	5 × 30°	c	c	c
			7 × 60°			
			4 × 90°			
		**Zone 7**	**Zone 8**	**Zone 9**	**Zone 10**	**Die**
		200	200	190	190	190
		7 × 60°	c	4 × 60°	c	-
		6 × 90°		4 × 90°		

## Data Availability

Data is contained within the article.

## Abbreviations

The following abbreviations are used in this manuscript:

FRC	Fibre-reinforced composites
PP	Polypropylene
GF	Glass fibre
MFR	Melt flow rate
IM	Injection moulding
C	Compounding
IFSS	Interfacial shear strength
